# Ranitidine-induced thrombocytopenia: A rare drug reaction

**DOI:** 10.4103/0253-7613.75676

**Published:** 2011-02

**Authors:** Amit V Bangia, Narendra Kamath, Vidushi Mohan

**Affiliations:** Department of Skin and VD, Kasturba Medical College, Mangalore, India

**Keywords:** Ranitidine, thrombocytocytopenia, adverse drug reaction

## Abstract

H_2_ antagonist ranitidine causing thrombocytopenia is a rare drug phenomenon. Here we present a case of 55 year old female of pustular psoriasis who presented with fever and vomiting. Patient. was started on roxithromycin, iv ondensetron, paracetamol and iv ranitidine. Complete blood count revealed neutrophilia with normal blood picture. However repeat investigations showed falling WBC and platelet count. After excluding other causes of pancytopenia we concluded that ranitidine was the cause for this atypical drug reaction, more so when the blood picture improved within 72 hrs of ranitidine withdrawal.

## Introduction

Thrombocytopenia due to H_2_ receptor antagonist is a rare but known phenomenon. Only 29 cases of H_2_ receptor antagonist (ranitidine and cimiteidine) induced thrombocytopenia have been reported so far. Though ranitidine and cimetidine belong to the same class of drugs, cross-reactivity between them is not conclusive. Platelet count typically falls from 75 to 80% of the normal value on exposure to the drug and returns to normal after drug withdrawal. Ranitidine is able to cause thrombocytopenia by an idiosyncratic reaction associated with an increase in platelet-induced immunoglobulins and its later clearance by the phagocytic system. The diagnosis of this critical condition is based on clinical suspicion and is a diagnosis of exclusion, probably life saving in critical patients.

## Case Report

A 55-year-old female, a case of pustular psoriasis, presented with multiple pustules over extremities, associated with fever and vomiting. Routine hemogram on admission revealed WBC count of 4900 cells/mm^3^ (N: 71, L: 23, E: 5, B: 1) and platelet count of 1.95 × 10^9^/dL. Hb was 10 g%. Patient was started on roxithromycin 150 mg BD, paracetomol 500 mg BD, IV ranitidine 50 mg BD and IV ondansetron. Blood investigations repeated after 2 days revealed thrombocytopenia with platelet count of 75 × 10^9^/dL, WBC count of 3000 cells/mm^3^ with neutropenia and Hb 9.4 g%. Important causes of pancytopenia like malaria, leptospirosis (this being an endemic area), dengue, autoimmune disorders, and sepsis were ruled out. Repeat blood picture showed further fall in platelet count to 30,000 × 10^9^/dL and WBC count to 1700 cells/mm^3^; however, there was no spontaneous bleeding. Bleeding time, clotting time and INR were normal. With other causes ruled out convincingly and the patient being clinically asymptomatic, it was concluded that a drug may be the possible cause of thrombocytopenia. Ranitidine-induced thrombocytopenia in critically ill patients has been reported. The drug was immediately withdrawn and after 48 hours a significant improvement in the blood picture was noticed with Hb 9.6 g%, WBC count of 4500 cells/mm^3^ and platelet count of 75 × 10^9^/dL. The patient had an uneventful recovery and was discharged a week later with normal blood counts.

## Discussion

Several drugs have been implicated in causing acute immunologically mediated thrombocytopenia.[1,2] There are three possible mechanisms by which a drug causes decrease in platelet count: failure of production by bone marrow, immune destruction and platelet aggregation in circulating blood.[3] Antibodies that bind to normal platelets in the presence of drug have been strongly implicated for drugs like cinchona, quinine and sulfa drugs.[4–7] However, in the case of H_2_ receptor antagonists, demonstration of drug associated antibodies (complement fixation, antiglobulin consumption tests and tests involving the release of platelets 11Cr or P173) is usually unsatisfactory[3] and diagnosis is based on clinical suspicion. In our case, to confirm the causality of ranitidine leading to thrombocytopenia, we applied the WHO-UMC system for standardized case causality assessment,[8] which suggested a “certain relationship” between ranitidine and thrombocytopenia though we could not subject the patient to a rechallenge. Drug-induced thrombocytopenia usually takes weeks or months to appear but may appear within 12 hours of drug intake in a sensitized individual.[9–11] Typically, the platelet count falls to 80% of the normal and thrombocytopenia may be associated with neutropenia and anemia. In our case, there was pancytopenia which significantly improved after withdrawal of ranitidine. Literature shows that this rare drug reaction due to H_2_ receptor antagonist (ranitidine) is more common in critically ill patients (patients admitted in ICU). Hence, precaution should be taken to put these patients on other medication for ulcer prophylaxis.

In conclusion, in cases of severe thrombocytopenia in critically ill patients, a pharmacological cause must be suspected, including H_2_ receptor blockers. Other alternate drug regimens for prophylaxis of stress ulcer should be considered.
